# Longitudinal Accuracy of Web-Based Self-Reported Weights: Results From the Hopkins POWER Trial

**DOI:** 10.2196/jmir.3332

**Published:** 2014-07-15

**Authors:** Gerald J Jerome, Arlene Dalcin, Janelle W Coughlin, Stephanie Fitzpatrick, Nae-Yuh Wang, Nowella Durkin, Hsin-Chieh Yeh, Jeanne Charleston, Thomas Pozefsky, Gail L Daumit, Jeanne M Clark, Thomas A Louis, Lawrence J Appel

**Affiliations:** ^1^Department of KinesiologyTowson UniversityTowson, MDUnited States; ^2^Division of General Internal MedicineJohns Hopkins University School of MedicineBaltimore, MDUnited States; ^3^Department of Psychiatry and Behavioral SciencesJohns Hopkins University School of MedicineBaltimore, MDUnited States; ^4^Department of Preventive MedicineRush University Medical CenterChicago, ILUnited States; ^5^Division of Endocrinology and MetabolismJohns Hopkins University School of MedicineBaltimore, MDUnited States; ^6^Department of BiostatisticsJohns Hopkins Bloomberg School of Public HealthBaltimore, MDUnited States

**Keywords:** self-report, weight loss, obesity, Internet

## Abstract

**Background:**

Websites and phone apps are increasingly used to track weights during weight loss interventions, yet the longitudinal accuracy of these self-reported weights is uncertain.

**Objective:**

Our goal was to compare the longitudinal accuracy of self-reported weights entered online during the course of a randomized weight loss trial to measurements taken in the clinic. We aimed to determine if accuracy of self-reported weight is associated with weight loss and to determine the extent of misclassification in achieving 5% weight loss when using self-reported compared to clinic weights.

**Methods:**

This study examined the accuracy of self-reported weights recorded online among intervention participants in the Hopkins Practice-Based Opportunities for Weight Reduction (POWER) trial, a randomized trial examining the effectiveness of two lifestyle-based weight loss interventions compared to a control group among obese adult patients with at least one cardiovascular risk factor. One treatment group was offered telephonic coaching and the other group was offered in-person individual coaching and group sessions. All intervention participants (n=277) received a digital scale and were asked to track their weight weekly on a study website. Research staff used a standard protocol to measure weight in the clinic. Differences (self-reported weight – clinic weight) indicate if self-report under (-) or over (+) estimated clinic weight using the self-reported weight that was closest in time to the clinic weight and was within a window ranging from the day of the clinic visit to 7 days before the 6-month (n=225) and 24-month (n=191) clinic visits. The absolute value of the differences (absolute difference) describes the overall accuracy.

**Results:**

Underestimation of self-reported weights increased significantly from 6 months (mean -0.5kg, SD 1.0kg) to 24 months (mean -1.1kg, SD 2.0kg; *P*=.002). The average absolute difference also increased from 6 months (mean 0.7kg, SD 0.8kg) to 24 months (mean 1.3, SD 1.8kg; *P*<.001). Participants who achieved the study weight loss goal at 24 months (based on clinic weights) had lower absolute differences (*P*=.01) compared to those who did not meet this goal. At 24 months, there was 9% misclassification of weight loss goal success when using self-reported weight compared to clinic weight as an outcome. At 24 months, those with self-reported weights (n=191) had three times the weight loss compared to those (n=73) without self-reported weights (*P*<.001).

**Conclusions:**

Underestimation of weight increased over time and was associated with less weight loss. In addition to intervention adherence, weight loss programs should emphasize accuracy in self-reporting.

**Trial Registration:**

ClinicalTrials.gov: NCT00783315; http://clinicaltrials.gov/show/NCT00783315 (Archived by WebCite at http://www.webcitation.org/6R4gDAK5K).

##  Introduction

The use of technology to support lifestyle-based treatment of obesity is commonplace in both research studies and commercial programs [1,2]. Specifically, self-monitoring of weight is a frequently recommended weight-loss strategy that can be facilitated by either websites or mobile phone apps [3]. Although self-monitoring of weight is a commonly identified, evidence-based strategy for weight loss, both technology-based and traditional paper-based cross-sectional studies indicate that individuals underestimate their weight and that the accuracy can vary for different demographic groups [4-8].

Few studies have examined the accuracy of self-reported weight over time. A 12-week weight management study (N=27) found self-reported weights recorded on a mobile phone were underestimated but were strongly correlated with weights taken in the clinic at both baseline and 12 weeks [9]. Results from a 6-month weight-loss trial (N=234) indicated that self-reported weights underestimated observed weights and those participants who lost more weight had more accurate self-report [10]. The accuracy of self-reported weight has not been examined in the context of a weight-loss study beyond 6 months.

The current study compared self-reported weight from a study website to clinic weights at 6-month and 24-month follow-up in the Hopkins Practice-Based Opportunities for Weight Reduction (POWER) trial, a three-arm randomized weight-loss trial with gender and race diversity [11,12]. The study also examined the association of accuracy of self-reported weight with the extent of weight loss and determined the rate of misclassification in achieving 5% weight loss when using self-reported compared to clinic weights as the follow-up weight.

## Methods

### Overview

The POWER trial at Hopkins was a randomized trial examining the effectiveness of two lifestyle-based weight-loss interventions (n=277) compared to a control group (n=138) among obese adult patients at six primary care practices [11,12]. Participants were at least 22 years of age, with a body mass index (BMI) ≥30 kg/m^2^ and at least one additional cardiovascular risk factor. An institutional review board approved the study and all participants provided informed consent. Detailed descriptions of intervention design, methods, and main results have been published [11,12].

### Intervention Summary

Intervention participants had a 5% weight-loss goal and access to a study website that included learning modules and tools for self-monitoring weight, caloric intake, and exercise. They also received a digital scale for home use and directions to (1) weigh themselves at the same time of day, in the same amount of clothing with the same scale, and (2) enter this weight on the study website at least weekly while trying to lose weight and daily during weight maintenance.

During the first 6 months, those in the Call-Center Directed intervention were offered 15 coaching calls and those in the In-Person Directed intervention were offered 21 groups and nine individual coaching sessions. From Month 7 to the end of the study, call-center participants were offered monthly calls and in-person participants were offered both individual and group sessions monthly.

### Primary Outcome

As previously reported, mean change in clinic measured weight (24 months – baseline) was −0.8 kg in the control arm, −4.6 kg in the call-center arm (*P*<.001 compared to control), and −5.1 kg in the in-person arm (*P*<.001 compared to control) [11]. There was no significant difference in weight loss between call-center directed and in-person directed arms. At 24 months, 37.9% (105/277) of the intervention participants achieved 5% weight loss.

### Measures

Demographics were self-reported at baseline. The number of completed coaching contacts and weight log-ins were recorded. Trained research staff, masked to intervention assignment, measured weights in the clinic at the randomization visit (baseline) and at the 6-month and 24-month follow-up visits. Following a standard protocol that included removing shoes and emptying pockets, weight was measured on a high-quality calibrated digital scale with the participant wearing light indoor clothes. Two weights were taken at each time point, and if needed, a third weight was taken to resolve any discrepancies. The assessment of weight during a clinic visit was independent of all intervention efforts. The accuracy of the clinic scales was verified annually by a third party.

Self-reported weight was based on the self-reported weight entered on the study website that was closest in time to the clinic weight and was within a window ranging from the day of the clinic visit to 7 days before the clinic visit. The intervention goal was to record a self-reported weight at least weekly, although the website allowed for daily self-report.

### Analytic Plan

Weights are reported in kilograms. Differences (self-reported weight – clinic weight) indicate under (−) or over (+) estimation of clinic weight. The absolute value of the difference (absolute difference) describes the overall accuracy. We regressed absolute difference on weight change and controlled for age, sex, race, baseline BMI, intervention arm, number of coaching contacts completed, number of weeks with a self-reported weight, and the difference in days between the self-reported and clinic weights.

In the main results paper, follow-up clinic weight was used to determine weight outcomes—both the amount of weight change and classifications of participants as either achieving or not achieving the study goal of 5% weight loss. In the current analyses, the same approach was used (ie, using clinic weights) to determine the gold standard for weight loss and subsequent classifications. We also used self-reported weights at 24-month follow-up to calculate an alternative weight loss and weight-loss classification. Misclassification refers to the alternative method (using self-reported follow-up) providing a different weight-loss classification compared to the gold standard (using clinic follow-up weight). We reported Cohen’s kappa comparing classifications using clinic weight compared to self-reported weight at follow-up to determine weight change. A Bland-Altman plot is presented comparing clinic and self-reported weights at 24 months. The intraclass correlation between self-reported and clinic weight at 24 months was also reported.

## Results

There were 277 participants in the active interventions. At 6 months, 28 did not have a self-reported weight within the target window and 24 were missing clinic weights resulting in 81.2% (225/277) with differences calculated. At 24 months, 83 did not have a self-reported weight within the target window and 13 were missing clinic weights resulting in 69.0% (191/277) with differences calculated.

Compared to those with missing data, the sample with differences calculated at 24 months had a higher percentage of men (*P*=.02), higher average contact completion (*P*<.001), and higher average number of self-reported weights (*P*<.001). Those with differences calculated at 24 months had an average age of 55 (SD 9.9) years, average BMI of 36 (SD 5.0) kg/m^2^, average weight of 104 (SD 18.7) kg, 59.2% were women (113/191), and 38.7% were black (74/191).

The average number of contacts was 14 (SD 3.4) at 6 months and 30 (SD 9.3) at 24 months. The average number of weeks with at least one weight log-in was 22 (SD 5.12) at 6 months and 71 (SD 26.4) at 24 months.

At 6 months, 61.8% (139/225) of self-reported weights were lower, 22.2% (50/225) were equivalent, and 16.0% (36/225) were higher than the clinic weights. At 24 months, 77.5% (148/191) of self-reported weights were lower, 9.4% (18/191) were equivalent, and 13.1% (25/191) were above the clinic weights. As seen in Table 1, the degree of underestimation increased from 6 months (mean −0.5kg, SD 1.0kg) to 24 months (mean −1.1kg, SD 2.0kg; *P*=.002). The average absolute difference also increased from 6 months (mean 0.7kg, SD 0.8kg) to 24 months (mean 1.3kg, SD 1.8kg; *P*<.001). Achieving the 5% weight loss goal was not associated with differences at 6 months (*P*=.24) or 24 months (*P*=.09). Participants who achieved 5% weight loss had lower absolute differences at 24 months (*P*=.01), but not at 6 months (*P*=.13) compared to those who did not meet this goal.

**Figure 1:**
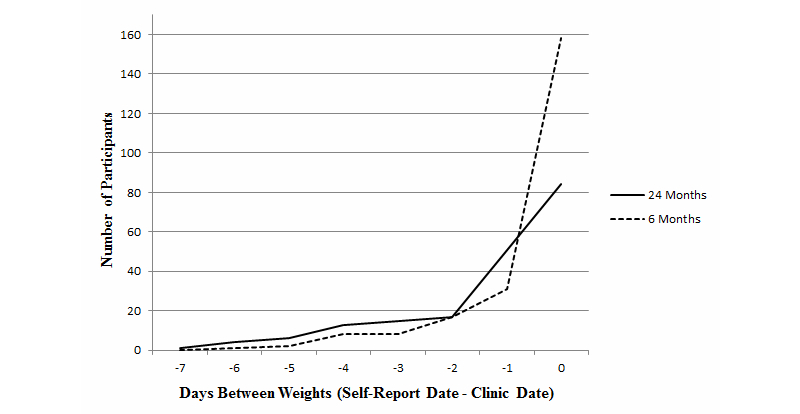
Time between self-reported and clinic weights at 6 and 24 months.

Figure 1 displays a distribution of the days between self-reported and clinic weights with a minimum score of −7 (self-reported weight was 7 days before the clinic weight) and a maximum score of 0 (self-reported and clinic weight were on the same day). At 6 months, 91.6% (206/225) of the self-reported weights were within 2 days of the clinic weight. At 24 months, 79.6% (152/191) of the self-reported weights were within 2 days of the clinic weight.

At 6 months, greater absolute difference between self-reported and clinic weight was associated with females, higher baseline BMI, less 6-month weight loss, fewer weeks with self-reported weights, and days between weights (Table 2). At 24 months, more weight loss and fewer days between weights were associated with smaller absolute differences. Significant associations among the independent and dependent variables were similar when examining differences rather than absolute differences as the outcome variable (data not shown).

When using self-reported weight at 24 months to calculate weight change from baseline clinic weight (see Table 3), there was 9% misclassification in achievement of weight-loss goal, 99% sensitivity, and 84% specificity compared to using clinic weights at 24 months. Cohen’s kappa was used to determine level of nonrandom agreement comparing the use of self-reported weight to the use of clinic weight at 24 months to determine weight loss classifications (κ=.82). Those included in Table 3 had greater 24-month weight loss (mean −6.0kg, SD 9.2kg) compared to those who did not have a self-reported weight within the target window at 24 months (mean −2.1kg, SD 5.3kg; *P*<.001).

**Figure 2:**
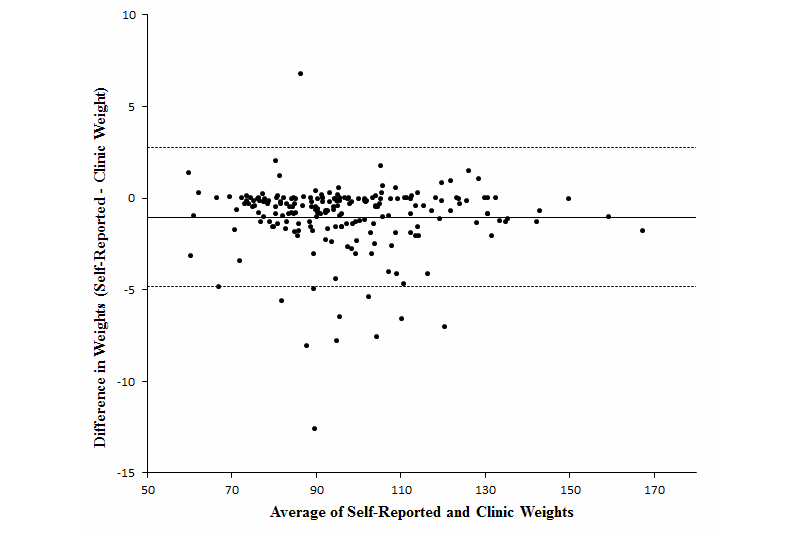
Differences in weights (self-reported – clinic) by the average of the two weights at 24 months.

Figure 2 is a Bland Altman Plot comparing self-reported and clinic weights at 24 months. A majority of the extreme differences between self-reported and clinic weights occur between 80 kg and 130 kg. The intraclass correlation comparing self-reported and clinic weights at 24 months was .99.

**Figure 3:**
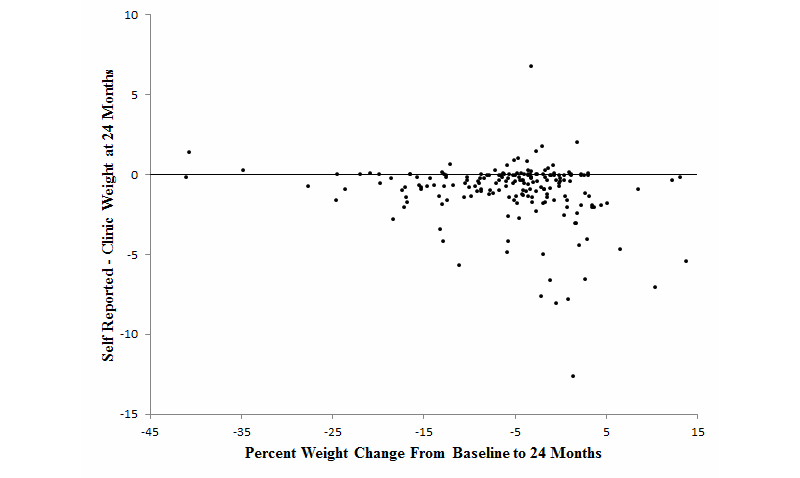
Accuracy of self-reported weight by percent weight change at 24 months.

Figure 3 displays differences between self-reported and clinic weights by the percent weight change at 24 months. Percent weight change was based on clinic weights. As seen in the figure, a majority of the self-reported weights were below compared to above the clinic weight. The spread of the differences increased as weight loss decreased.

**Table 1:** Difference in weights (self-reported – clinic) by attainment of 5% weight-loss goal at 6 and 24 months.

Difference	6 months			24 months		
	All (n=225)	Achieved 5% weight loss (n=112)	Did not achieve 5% weight loss (n=113)	All (n=191)	Achieved 5% weight loss (n=82)	Did not achieve 5% weight loss (n=109)
Difference^a^ in kg, mean (SD)	−0.5 (1.0)	−0.4 (0.8)	−0.6 (1.1)	−1.1 (2.0)	−0.8 (1.2)	−1.2 (2.4)
Absolute difference^b^ in kg, mean (SD)	0.7 (0.8)	0.6 (0.7)	0.8 (1.0)	1.3 (1.8)	1.0 (1.1)	1.5 (2.2)

^a^Difference (self-reported – clinic weight).

^b^Absolute difference = |self-reported weight – clinic weight|.

**Table 2:** Association of absolute difference in weights (self-reported – clinic) with weight change, demographic characteristics, and participation at 6 and 24 months.

Independent variable	Absolute difference^a^			
	6 months		24 months	
	B (SE)^b^	*P*	B (SE)^b^	*P*
Weight change^c^ in kg	.04 (0.01)	<.001	.05 (0.02)	.01
Female	.38 (0.11)	.001	.19 (0.28)	.50
Black	.06 (0.11)	.58	.53 (0.28)	.06
Age	.003 (0.01)	.55	.02 (0.01)	.27
Body Mass Index (baseline)	.03 (0.01)	.02	.03 (0.03)	.31
Intervention arm	−.10 (0.11)	.36	−.45 (0.27)	.10
Coach contacts completed	−.00 (0.02)	.64	.02 (0.02)	.44
Self-reported weight, frequency	.04 (0.01)	.01	.00 (0.01)	.81
Days between weights	−.24 (0.04)	<.001	−.24 (.08)	.003

^a^Absolute difference = |self-reported weight – clinic weight|.

^b^B=beta coefficient, SE=standard error; adjusted for all variables listed.

^c^Weight change = follow-up clinic weight – baseline clinic weight.

**Table 3:** Weight loss goal attainment at 24 months based on final weight from clinic and self-reported weight (n=191).

Self-reported weight		Clinic weight	
		Achieved 5% weight loss, n (%)	Did not achieve 5% weight loss, n (%)
Achieved 5% weight loss		93 (48.7)	16 (8.4)
Did not achieve 5% weight loss		1 (0.5)	81 (42.4)

## Discussion

### Principal Findings

To our knowledge, this is the first study to document decreases in accuracy of self-reported weights over long-term follow-up. Specifically, participants’ self-report resulted in a modest, yet significant underestimation of weight consistent with previous findings from cross-sectional studies and weight-loss trials [4,5,9,10]. The magnitude of underestimation doubled between Months 6 and 24. Moreover, weight loss was positively associated with accuracy; those who achieved the 5% weight-loss goal had more accurate self-reports. These results are congruent with findings from a study with 6 months of follow-up [10]. Strengths of the current analyses include a relatively large sample of obese adults with gender and ethnic diversity and a long duration of follow-up (ie, 24 months).

There are a number of possible sources of variation between self-reported weight and clinic weights. There was likely a difference in the accuracy of the scales provided to participants and the clinic scale that had annual accuracy verification. Another possible source of variation was diurnal weight fluctuations and other factors associated with the time lag between weights (eg, actual weight change, menstrual cycle). However, the regression analyses found a significant association among absolute difference and weight change even after controlling for length of time between measures. Although the participants were encouraged to weigh themselves under the same conditions experienced in the clinic, there may have been differences in procedures (eg, amount of clothing, placing scale on hard surface) associated with differences.

One alternative to manually entering self-reported weights on a website is the use of scales with wireless connections to a computer that can automatically upload data. However, little is known about the accuracy of this method and sources of variation may be similar to those outlined above. Pronk et al demonstrated that participants can improve the accuracy of self-reported weights with regular feedback [13]. Improvements in the accuracy of self-reporting may help individuals with long-term adherence to lifestyle-based programs.

Although short-term fluctuation in weight is expected, the extent to which small changes and the tracking of subtle trends impacts weight loss is unclear. During weight-loss maintenance, it can be difficult to reverse the trajectory of even minor weight gains [14]. Less accurate self-assessment could create challenges in identifying small changes. It is possible that the tendency to underestimate weight and increased error over time create additional barriers to long-term weight-loss efforts.

The underestimation of clinic weights in the current study was modest, and the sensitivity and specificity appears acceptable. Other studies have reported strong correlations between self-reported and clinic weights and describe the differences between self-reported and clinic weights as relatively small [9,13]. However, researchers should be cautious in using self-reported weight as a clinical outcome. The current results are consistent with a previous report of modest but significant differences between weight change calculated with self-reported versus clinic values [10]. Moreover, the generalizability of the findings are limited by the completer analysis. In the current study, 95% of the sample had 24-month clinic weights, yet only 69% had both self-reported and clinic weights. Those with self-reported weight had three times the weight loss compared to those without self-reported weights at 24 months, and this is concordant with previous reports [10].

### Conclusions

Although it is not clear if accurate self-weighing facilitated weight loss or if weight loss encouraged more exacting self-assessment, those in weight-loss programs should be aware of the tendency for decreased accuracy of self-reported weight over time and the association of accurate self-report with achievement of weight-loss goals. Increased emphasis on accurate self-weighing may be an important addition to lifestyle-based weight-loss programs.

## Abbreviations

BMI: body mass index
POWER: Practice-Based Opportunities for Weight Reduction trial
